# Comparative efficacy of oral dutasteride and low-, medium-, and high-dose oral minoxidil: a six-month prospective trichoscopic and clinical study

**DOI:** 10.3389/fmed.2026.1751116

**Published:** 2026-02-16

**Authors:** M. Larios-Cárdenas, D.G. Quiñones-Hernández, M. León Quintero-Loreto, F. Grover-Páez, J.A. Moreno-Alanis, R. Quiñones-Hernández, E.R. Calderón-Quiroz, V. Carrillo-Manguart, M. Alcocer-Salas, S. González-Ruíz, M.G. Ramos-Zavala, D. Cardona-Muller, L.E. Sánchez-Dueñas

**Affiliations:** 1 Dermatologic Institute of Jalisco “Dr. José Barba Rubio”, University of Guadalajara, Guadalajara, Mexico; 2 Laboratory of Vascular Mechanics, Institute of Experimental and Clinical Therapeutics, University Center of Health Sciences, University of Guadalajara, Guadalajara, Mexico; 3 University of Guadalajara, Guadalajara, Mexico; 4 Hospital “Dr. Valentín Gómez Farías” ISSSTE, University of Guadalajara, Guadalajara, Mexico

**Keywords:** alopecia, clinical outcomes, dutasteride, oral minoxidi, trichoscopy

## Abstract

**Introduction:**

Androgenetic alopecia is a common progressive hair loss disorder that affects quality of life and often prompts long-term medical management. Oral minoxidil and oral dutasteride are widely used treatments, yet comparative data on their early clinical and microscopic effects remain limited. This study examined six-month changes in clinical severity and regional hair characteristics in men receiving different doses of oral minoxidil or oral dutasteride.

**Methods:**

Men with clinically confirmed androgenetic alopecia received oral dutasteride 0.5 milligrams daily or oral minoxidil at doses of 1, 2.5, or 5 milligrams daily, with treatment selection guided by clinical judgment and patient preference. Standardized clinical staging and trichoscopic imaging of the fronto-parietal, temporal, and occipital scalp regions were performed at baseline, 3 months, and 6 months. Outcomes included changes in clinical severity and region-specific measures of hair density, hair shaft diameter, and follicular unit composition.

**Results:**

Sixty-seven participants were enrolled, and forty-six completed the six-month assessment. Across the oral minoxidil groups, improvements in clinical severity scores were observed, whereas no statistically significant change was detected in the oral dutasteride group over the same period. Trichoscopic changes were generally modest across all treatment regimens, with no group demonstrating significant increases in total hair density. Selective, region- and dose-specific structural changes were noted, including a higher proportion of thicker hairs in the fronto-parietal region with oral dutasteride and increased hair shaft diameter in the occipital region with low-dose oral minoxidil. However, these findings were limited in scope and did not correspond to broad improvements across trichoscopic parameters.

**Discussion:**

In this exploratory, non-randomized clinical trial, oral minoxidil was associated with short-term improvements in clinical severity, while oral dutasteride did not demonstrate measurable early clinical change within the six-month follow-up. Trichoscopic findings were limited and did not consistently parallel clinical outcomes, suggesting that early therapeutic effects in routine practice may be more readily captured by clinical staging than by regional microscopic assessment. These observations should be interpreted cautiously given the study design and sample size, and they warrant confirmation in larger, randomized studies with longer follow-up.

## 1 Introduction

Androgenetic alopecia (AGA) is the most common cause of hair loss and has significant clinical and emotional effects. It is characterized by the gradual miniaturization of terminal hair follicles beginning after puberty and becomes more prevalent with age, affecting most men and approximately half of women by age 70, with the highest rates reported in Caucasian populations (1). Beyond cosmetic concerns, AGA has measurable impact on quality of life. A meta-analysis of nearly 8,000 individuals found moderate impairment in dermatology- and hair-specific quality-of-life scores, highlighting the psychological and social burden of the condition, even in the absence of a clear association with clinical depression (2).

At the biological level, AGA results from an interplay of genetic susceptibility and androgen-driven processes. Susceptible follicles exhibit altered local enzyme activity, specifically higher 5α-reductase and lower aromatase expression, facilitating increased conversion of testosterone to dihydrotestosterone (DHT). Elevated DHT influences dermal papilla cell signaling and shifts the follicular environment toward inhibitory pathways involving mediators such as TGF-*β*, DKK-1, and IL-6, which collectively contribute to progressive follicle miniaturization (3). These mechanisms are most pronounced in areas with heightened androgen sensitivity, explaining the characteristic male-pattern distribution in the fronto-parietal, temporal, and vertex regions. In contrast, occipital follicles generally show relative androgen independence (4).

Therapeutic strategies frequently target the androgen pathway. Dutasteride, which inhibits both type I and II 5α-reductase isoenzymes, offers deep suppression of DHT and is more potent in this regard than finasteride (5). Clinical experience supports its utility; in one real-world cohort, a substantial majority of men receiving dutasteride showed clinical improvement, and higher doses were associated with more pronounced benefit (6). The tolerability profile is generally acceptable. Reported adverse effects, including reduced libido, gastrointestinal symptoms, pruritus, and erectile dysfunction, occur in a minority of patients, and low-dose regimens appear particularly well-tolerated (5–7).

Oral minoxidil provides an alternative mechanism of action. As a potassium-channel opener, it enhances scalp blood flow and may promote follicular recovery through vascular and anti-inflammatory effects, including influences on VEGF and Wnt/*β*-catenin signaling (8). Evidence supports its effectiveness in both men and women at appropriately selected doses. In women, low daily doses yield results comparable to topical formulations, while in men, higher doses have produced strong improvements in controlled studies (7).

Choosing between dutasteride and oral minoxidil can be challenging given their distinct mechanisms and response patterns. Some comparative analyses suggest that dutasteride may enhance overall hair count more effectively, whereas minoxidil may produce more pronounced gains in terminal hairs, underscoring the complementary nature of their effects (9). Molecular variability further complicates treatment selection; differences in expression of 5α-reductase isoenzymes among individuals may influence responsiveness to DHT-modulating therapies (5).

Trichoscopy has emerged as a central tool in evaluating therapeutic responses and in diagnosing AGA more broadly. Characteristic findings include marked hair shaft variability in thickness, one of the most sensitive trichoscopic markers of the condition, while other features such as the peripilar sign occur less frequently but have high specificity and can strongly support the diagnosis (9, 10). Beyond diagnostic utility, trichoscopy offers a non-invasive method for monitoring follicular changes over time; however, the extent to which early trichoscopic changes parallel short-term clinical improvement remains incompletely defined.

The present study was therefore designed to evaluate early clinical and trichoscopic responses over 6 months in patients with AGA treated with oral dutasteride or low-, medium-, and high-dose oral minoxidil. The primary objective was to assess changes in clinical severity, while secondary objectives included the analysis of region-specific trichoscopic parameters, including hair density, shaft diameter, and follicular unit composition, across treatment regimens.

## 2 Methods

This was an open-label, multi-arm, non-randomized clinical trial conducted over 6 months to evaluate the efficacy and safety of four oral therapeutic regimens used in the management of androgenetic alopecia. Participants were assessed at three predefined time points (baseline, month three, and month six) and underwent standardized clinical examinations, trichoscopic imaging, patient-reported outcome assessments, and safety evaluations. The study was conducted at a dedicated dermatology and hair disorders clinic, and all procedures followed a uniform operational protocol. Ethical approval was obtained from the Comité de Ética en lnvestigación del Centro de Estudios de lnvestigación Básica y Clínica S.C. (CECEIBAC) review board (20.01.205), and all participants provided written informed consent prior to enrollment.

Eligible participants were adults with a clinical diagnosis of androgenetic alopecia, confirmed through medical history, physical examination, and trichoscopic findings. Individuals with Hamilton–Norwood stages I to VII were included as long as they expressed willingness to comply with treatment and follow-up visits. Participants were excluded if they had used topical or systemic treatments known to affect hair growth within the preceding 6 months, had any concomitant scalp conditions that interfered with evaluation, or were taking medications that could influence hair biology. Additional exclusions included contraindications to oral minoxidil or dutasteride, uncontrolled cardiovascular disease, or hypotension.

Participants were assigned to one of four treatment regimens based on clinical suitability and shared decision-making between physician and patient. These regimens included oral dutasteride 0.5 mg daily, high-dose oral minoxidil 5 mg daily, medium-dose oral minoxidil 2.5 mg daily, or low-dose oral minoxidil 1 mg daily. Each participant was instructed to take the medication once daily at the same time, and no concurrent hair loss treatments were permitted during the study period to avoid confounding effects.

Trichoscopic assessments were conducted using a standardized imaging protocol to ensure high reproducibility across visits. First, clinical overview photographs of the scalp were obtained using a medical-grade camera system under controlled lighting and positioning. Subsequently, dermoscopic imaging was performed with a handheld pistol-type dermatoscope equipped with an integrated camera (FotoFinder Studio®, Tricholab Software v2.0.63.0 1.1). For each anatomical region, trichoscopic images were acquired using a predefined scalp mapping approach with measurements obtained at 1-cm intervals. In the fronto-parietal region, imaging was performed along the midline, starting at the frontal hairline (defined as the point at which terminal hair growth begins) and progressing posteriorly toward the vertex. In the temporal region, measurements were obtained approximately two finger widths above the superior attachment of the ear, beginning at the temporal hairline and progressing inferiorly toward the nape of the neck, with acquisition points spaced 1 cm apart. In the occipital region, imaging was centered on the occipital eminence, with two measurement points acquired to the right and two to the left of the midline, each separated by 1 cm and progressing superiorly toward the vertex.

**Figure 1:**
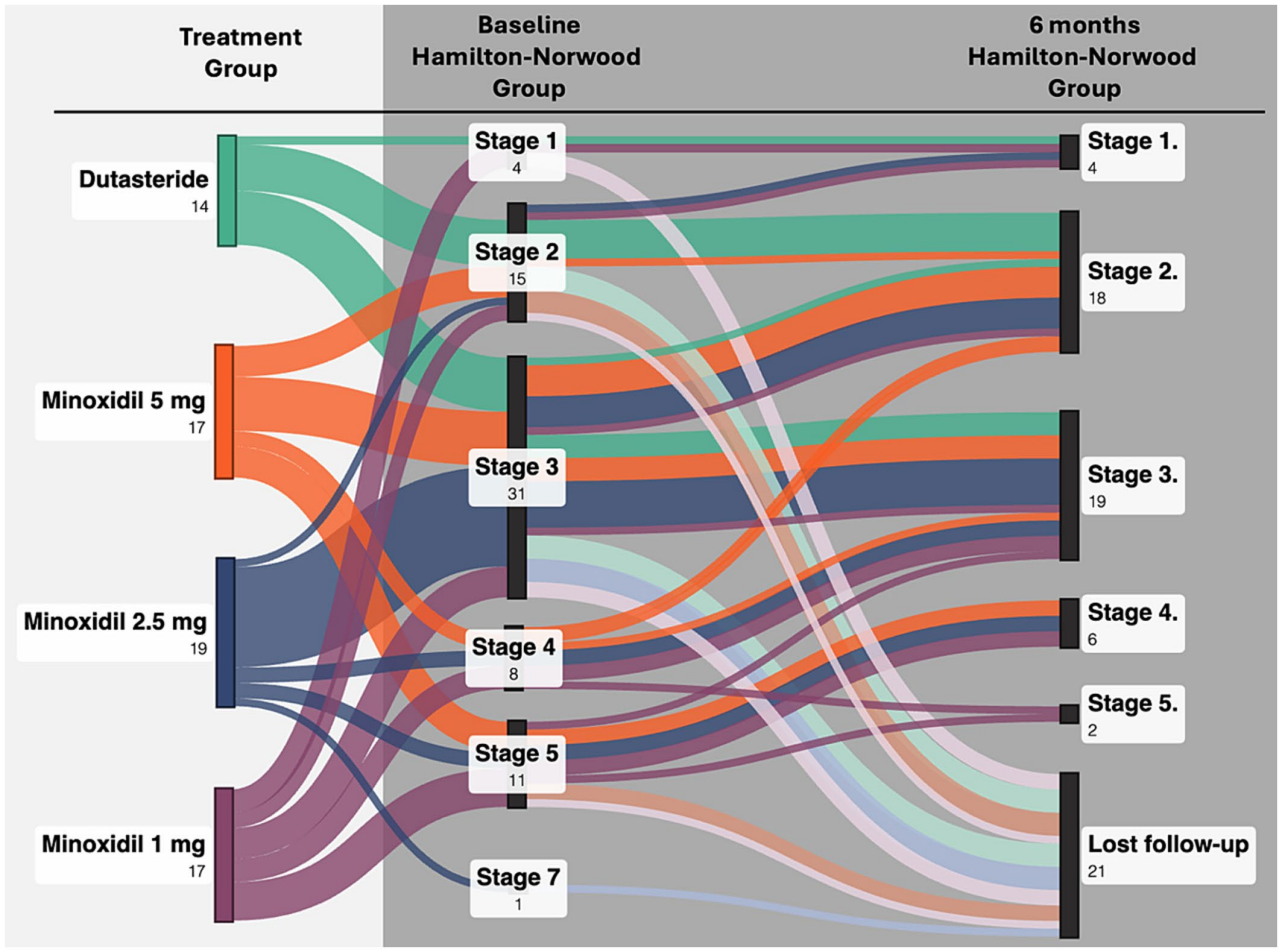
Transitions in Hamilton–Norwood stage from baseline to month 6 across treatment groups.

Within each region, seven quantitative trichoscopic parameters were extracted: total hair density (hairs/cm^2^), proportion of thick hairs (>50 μm), proportion of thin hairs (30–50 μm), and proportion of miniaturized or vellus hairs (<30 μm), along with mean hair shaft caliber and follicular unit composition. All images were acquired using consistent magnification and illumination settings. Measurements were automatically generated by the device’s AI-supported analysis software. A schematic reference of the scalp mapping and measurement locations is provided in Figure 1.

Clinical severity of androgenetic alopecia was assessed using the Hamilton–Norwood classification at baseline, month three, and month six. These evaluations provided a global, ordinal assessment of disease severity and were used to complement quantitative trichoscopic outcomes. Clinical grading was performed independently by two dermatologists who were blinded to treatment allocation and study time points, with discrepancies resolved by consensus. Participants were systematically queried about adverse events at each visit, and treatment was adjusted or discontinued when clinically indicated.

Statistical analyses were performed using R (version 2023.12.1 + 402). Continuous variables were analyzed using means and standard deviations or medians and interquartile ranges, depending on data distribution. Longitudinal changes from baseline to month three and month six were examined using paired t-tests or Wilcoxon signed-rank tests, while between-group differences were evaluated with the Kruskal–Wallis test. Statistical significance was defined as a *p*-value below 0.05.

## 3 Results

### 3.1 Participant characteristics

A total of 67 participants were enrolled in the study. Baseline androgenetic alopecia severity ranged from Hamilton–Norwood stage I to VII, with stage III being the most common presentation. Participants were evenly distributed across the four treatment regimens, and baseline demographic and clinical characteristics were comparable between groups.

Participants were distributed evenly across the four treatment regimens: 14 in the dutasteride 0.5 mg group (21%), 17 in the minoxidil 5 mg group (25%), 19 in the minoxidil 2.5 mg group (28%), and 17 in the minoxidil 1 mg group (25%). Detailed baseline characteristics are summarized in Supplementary Table 1. No statistically significant differences were observed between treatment groups for body mass index (*p* = 0.12) or Hamilton–Norwood stage distribution (*p* = 0.06), indicating balanced cohorts at study entry.

Of the 67 enrolled participants, 46 (69%) completed the six-month evaluation, while 21 (31%) were lost to follow-up. Completion rates did not differ significantly across treatment groups (*p* = 0.58). Follow-up patterns in this cohort likely reflect real-world esthetic practice, where long-term retention can be challenging due to the elective nature of treatment. While treatment adjustment or discontinuation was permitted for safety reasons, no clear pattern of adverse event–related discontinuation was observed; losses to follow-up were mainly due to non-attendance at scheduled visits. Baseline demographic and trichoscopic characteristics stratified by treatment group and completion status are presented in Supplementary Table 2, and participant flow from enrollment through baseline assessment and six-month outcomes across treatment groups is summarized in Supplementary Figure 1.

### 3.2 Primary outcomes

#### 3.2.1 Clinical outcomes

**Figure 2:**
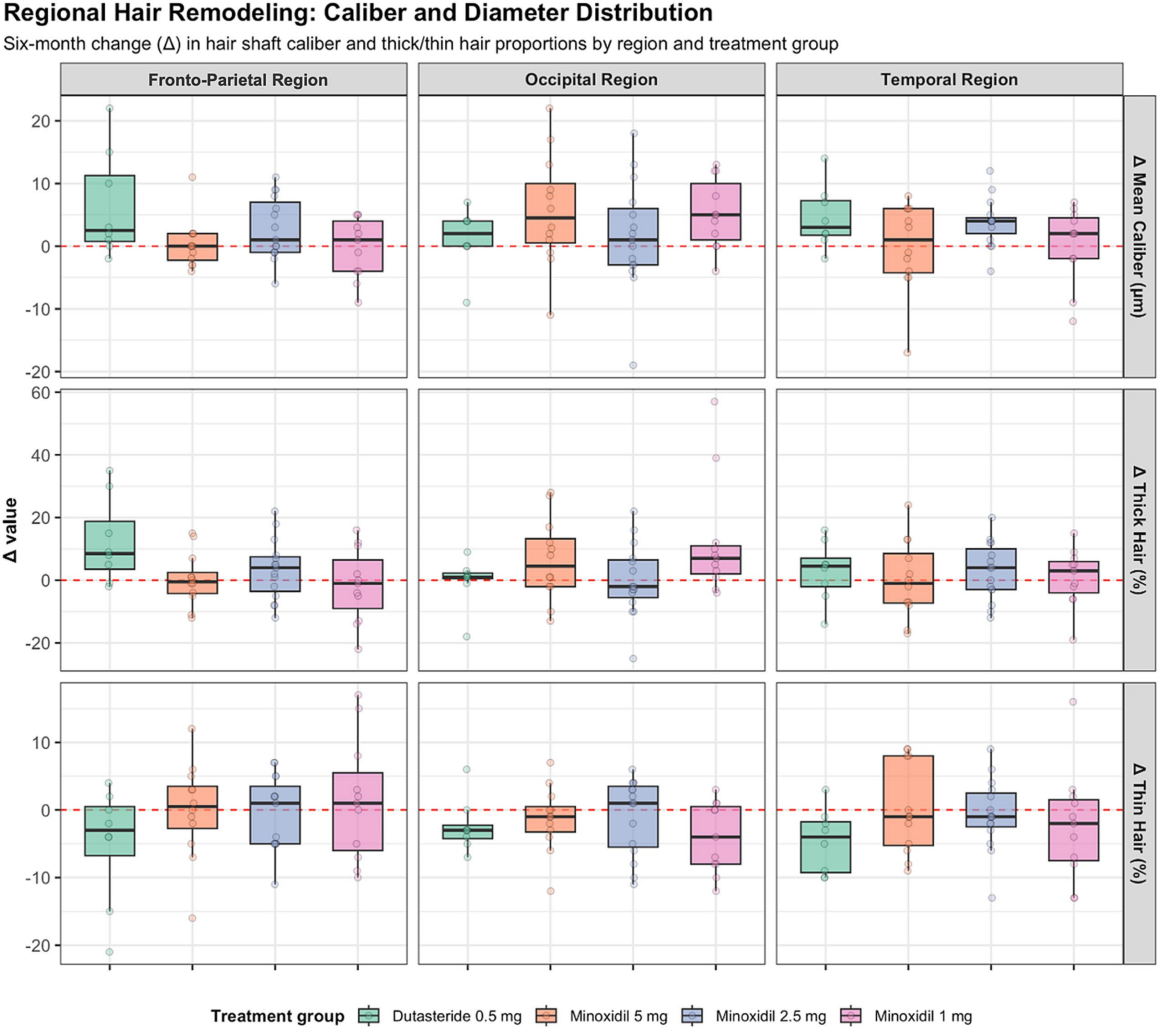
Regional changes in hair shaft caliber and thickness across treatment groups.

Clinical severity assessed using the Hamilton–Norwood classification, showed significant improvement over time in all three oral minoxidil groups. Participants receiving minoxidil 5 mg, 2.5 mg, and 1 mg demonstrated progressive reductions in clinical severity across follow-up visits (*p* < 0.008, *p* < 0.003, and *p* = 0.03, respectively). These findings indicate that minoxidil produced measurable global improvement over the six-month period. In contrast, the dutasteride group did not show a statistically significant change in classification severity (*p* = 0.36), suggesting limited clinical progression within the study timeframe. Figure 2 illustrates the transitions in Hamilton–Norwood stage from baseline to month 6 across treatment groups.

### 3.3 Secondary quantitative trichoscopy-derived outcomes

#### 3.3.1 Baseline trichoscopy-derived parameters

Baseline trichoscopic measurements were similar across all four treatment groups in the fronto-parietal, occipital, and temporal regions. In the fronto-parietal scalp, median total density ranged from 158.0 to 178.0 hairs per square centimeter, the proportion of thick hairs ranged from 47.0 to 63.5 percent, and mean shaft diameter ranged from 52.0 to 59.0 micrometers. The occipital region showed comparable values, with median density between 156.0 and 175.0 hairs per square centimeter and shaft diameters between 63.0 and 68.0 micrometers. Temporal density ranged from 115.5 to 135.0 hairs per square centimeter with shaft diameters between 64.0 and 67.0 micrometers. Follicular unit distributions, including single-, double-, and triple-hair follicular units, also overlapped across regimens.

Kruskal–Wallis tests indicated no significant between-group differences at baseline. In the fronto-parietal region, *p* values ranged from 0.23 to 0.87. In the occipital and temporal regions, p values ranged from 0.15 to 0.93 and 0.07 to 0.97, respectively. These findings indicate that all four groups were trichoscopically comparable at baseline.

#### 3.3.2 Fronto-parietal region

**Figure 3:**
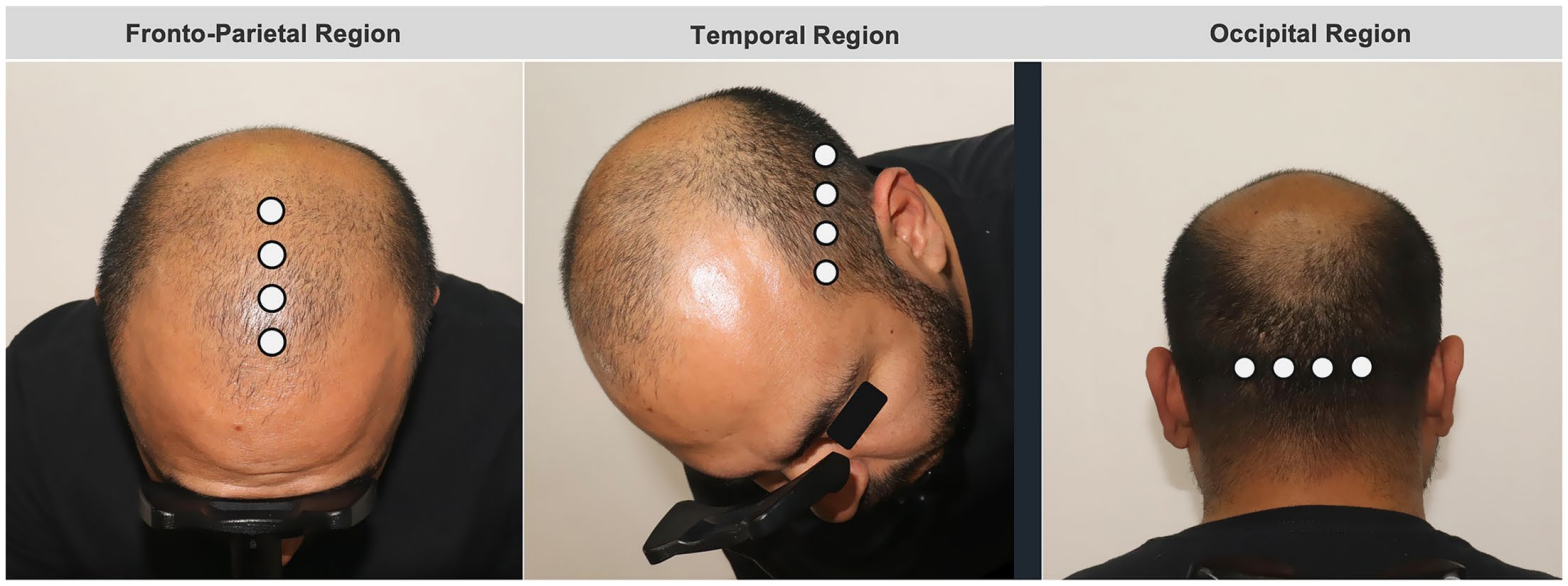
Schematic representation of scalp mapping and trichoscopic measurement locations.

Secondary trichoscopic outcomes in all regions are summarized in Figure 3. Overall, six-month changes in trichoscopy-derived structural parameters were modest across treatment groups, and no regimen demonstrated a significant improvement in total hair density. The most consistent within-group signal was observed with dutasteride, which showed a significant increase in the proportion of thicker hairs (*p* = 0.04) and a corresponding reduction in thin hairs (*p* = 0.05). Changes in mean shaft caliber were small and did not reach statistical significance in any group. Between-group comparisons did not identify significant differences in changes in shaft diameter (*p* = 0.17), thick-hair proportion (*p* = 0.12), thin-hair proportion (*p* = 0.66), or follicular unit composition (all *p* > 0.27).

#### 3.3.3 Occipital region

The occipital region exhibited relative structural stability over 6 months, with only small changes in total hair density across all regimens. Trichoscopy-derived parameters, including hair shaft caliber and thick and thin hair proportions, showed minimal variation and were centered around zero across treatment groups. A modest within-group increase in mean shaft diameter and thicker-hair proportion was observed in participants receiving minoxidil 1 mg (both *p* = 0.02); however, all other within-group comparisons were non-significant. Between-group analyses confirmed the absence of meaningful differences in shaft thickness (*p* = 0.37), thicker-hair proportion (*p* = 0.20), thin-hair proportion (*p* = 0.39), or follicular unit composition. Overall, the occipital region demonstrated limited biological variability and minimal treatment-associated divergence.

#### 3.3.4 Temporal region

The temporal region demonstrated the greatest heterogeneity in trichoscopic outcomes. No regimen produced a significant gain in total hair density, and participants receiving minoxidil 1 mg experienced a significant decline (median −13 hairs/cm^2^, *p* = 0.03). Modest increases in hair shaft caliber were observed across regimens, reaching statistical significance only with minoxidil 2.5 mg (median +4 μm, *p* < 0.003). Changes in thick and thin hair proportions were generally small, with a significant reduction in thin hairs observed only in the dutasteride group (*p* = 0.05), and no significant between-group differences.

In contrast to other regions, follicular unit architecture differed between treatments in the temporal region. Changes in single-hair follicular units showed significant between-group differences (*p* = 0.01), as did changes in double-hair follicular units (*p* = 0.04), whereas triple-hair units demonstrated minimal, non-significant variation. Overall, the temporal region appeared most vulnerable to structural decline, particularly at lower minoxidil doses, and exhibited greater interindividual variability than the fronto-parietal and occipital regions.

Across all scalp regions, trichoscopy-derived changes were generally modest and variable, with substantial overlap between treatment groups. Table 1 summarizes six-month changes (*Δ*) in total hair density across scalp regions and treatment groups, with the corresponding scalp mapping and measurement locations illustrated in Supplementary Figure 2. Figure 3 illustrates the distribution and heterogeneity of six-month changes in hair shaft caliber and thick and thin hair proportions, while statistical testing highlights only limited within-group effects and few region-specific between-group differences.

**Table 1:** Six-month change (Δ) in total hair density across regions and treatment groups.

Region	Treatment group	Median Δ (IQR)	Mean Δ ± SD	Within-group *p*	Effect size (*r*)	Between-group *p*
Fronto-parietal	Dutasteride 0.5 mg	−14.5 (−28.0 to 10.8)	−9.6 ± 25.0	0.291	0.40	0.236
	Minoxidil 5 mg	13.5 (−0.5 to 26.5)	12.7 ± 23.6	0.099	0.49	
	Minoxidil 2.5 mg	−8.0 (−15.0 to 10.0)	−1.7 ± 35.1	0.670	0.12	
	Minoxidil 1 mg	0.0 (−9.0 to 8.0)	5.1 ± 41.5	1.000	0.01	
Occipital	Dutasteride 0.5 mg	4.5 (−11.8 to 13.8)	0.6 ± 23.7	0.945	0.05	0.870
	Minoxidil 5 mg	3.5 (−12.5 to 15.5)	−3.9 ± 30.3	0.906	0.05	
	Minoxidil 2.5 mg	−8.0 (−21.5 to 10.0)	−7.6 ± 23.2	0.315	0.26	
	Minoxidil 1 mg	−9.0 (−29.0 to 22.5)	−9.5 ± 36.9	0.476	0.23	
Temporal	Dutasteride 0.5 mg	−2.0 (−18.2 to 2.5)	−9.4 ± 18.7	0.353	0.32	0.106
	Minoxidil 5 mg	−2.0 (−10.2 to 7.8)	−1.8 ± 12.3	0.695	0.12	
	Minoxidil 2.5 mg	0.0 (−7.0 to 10.0)	−0.5 ± 20.8	0.802	0.07	
	Minoxidil 1 mg	−13.0 (−42.0 to −6.0)	−20.9 ± 24.9	**0.032**	0.68	

Bold type indicates statistical significance (*p* < 0.05).

## 4 Discussion

This study examined the six-month effects of oral dutasteride and three doses of oral minoxidil on trichoscopic parameters and clinical outcomes in men with androgenetic alopecia. Baseline characteristics were balanced across groups, and changes in trichoscopic parameters were modest, with no treatment showing significant gains in total hair density. Structural changes were observed in specific regions and dose groups, but these shifts were limited and were not accompanied by widespread improvements in microscopic metrics. In comparison, changes in clinical severity were more apparent in the oral minoxidil groups, while remaining largely unchanged in the dutasteride group. Taken together, these observations suggest that, over short follow-up periods, potential treatment-related changes may be more readily captured through clinical staging than through trichoscopic assessment, particularly in the fronto-parietal scalp.

Clinical improvement across all three minoxidil doses in our cohort aligns with prior research showing that changes in hair caliber and the terminal-to-vellus ratio strongly influence perceived hair volume and clinical staging in androgenetic alopecia (11, 12). Studies comparing oral and topical minoxidil have also shown that oral formulations can produce similar or slightly greater clinical changes in specific regions, particularly the vertex, even when differences in hair density are modest at early follow up (13). These findings are consistent with the pattern observed in our results, where clinical severity improved even in the absence of large gains in total density. In contrast, dutasteride did not produce significant clinical improvement over 6 months in our sample, although meta-analytic evidence has shown that low dose dutasteride can generate substantial increases in total hair count at 24 weeks and may outperform finasteride and several minoxidil formulations when evaluated through global hair count endpoints (14). Several region- and dose-specific trichoscopy-derived findings observed in this study did not demonstrate consistent alignment with expected biological patterns. Given the small subgroup sizes, baseline regional variability, and early follow-up period, these observations may reflect measurement variability rather than true differential treatment effects and should therefore be interpreted cautiously.

Mechanistic differences may also help explain why clinical improvement occurred consistently with minoxidil and not with dutasteride within the six-month timeframe. Minoxidil has well described vascular and proliferative effects that can lead to early changes in hair caliber and follicular activity, factors that contribute meaningfully to the perception of volume and clinical staging (11). These early shifts may not always manifest as clear gains in total density but can still produce visible improvements, as noted in both randomized trials and observational studies (12, 13). Dutasteride, in contrast, acts through androgen suppression and may require longer exposure before measurable structural changes become evident. This slower timeline has been reflected in comparative analyses, in which the most pronounced gains with dutasteride are typically observed with follow-up approaching 1 year (14). Additional evidence indicates that combination therapies involving minoxidil can produce stronger density responses than monotherapy, suggesting that hair cycle modulation through multiple pathways may be necessary for early measurable gains in density (15).

A dissociation between clinical and trichoscopic outcomes was evident in our study, with clinical severity improving across all minoxidil groups despite minimal changes in density and shaft caliber. Prior work has shown that patient-reported improvement correlates with changes in density but not with changes in hair width, and that density differences distinguish improved, stable, and worsened groups, while width does not (16). The absence of significant density gains in our cohort, combined with measurable clinical improvement, indicates that clinical classification and trichoscopic parameters may capture different aspects of early treatment response. Similar fronto-parietal and occipital hair density in Norwood grade III AGA underscores the limitations of hair density as a standalone metric in early disease, where follicular miniaturization and shaft caliber changes predominate over follicle loss. Standardized regional sampling may also miss the most affected micro-areas, which could contribute to both comparable density measurements and the limited trichoscopic changes observed after treatment.

The limited early trichoscopic changes observed in this cohort underscore the importance of carefully selecting monitoring tools that are appropriate for detecting early or short-term changes in androgenetic alopecia. Prior evidence indicates that trichoscopy offers high diagnostic utility in pattern hair loss and may outperform traditional methods such as the trichogram, particularly in early-stage female androgenetic alopecia, where variability in hair shaft diameter has been shown to provide a useful diagnostic signal (17, 18). These findings suggest that clinical follow-up should prioritize trichoscopic parameters that reflect shaft diversity or follicular miniaturization rather than relying solely on density-based measures. Incorporating standardized trichoscopic evaluation into routine visits may improve the detection of treatment-related changes and enhance clinical decision-making, especially when visible improvement precedes measurable increases in density.

The divergence between clinical and trichoscopic outcomes in the present study underscores the need for more objective and reproducible tools for assessing therapeutic response in androgenetic alopecia. Emerging AI-based grading systems offer a potential framework for improving consistency, as demonstrated by recent work showing high precision and reliability in identifying areas of hair loss using quantitative area ratio metrics as alternatives to conventional classification scales (19).

Nonetheless, AI-derived trichoscopic parameters are subject to inherent technical constraints. Automated detection algorithms may be challenged in settings of closely clustered hairs, complex follicular architecture, or variable image quality, which could limit sensitivity for detecting subtle changes in density or shaft caliber. These considerations highlight the need for cautious interpretation of quantitative outputs. When appropriately contextualized, AI-supported image analysis may still help overcome some limitations of subjective staging systems and provide more standardized endpoints for future clinical trials.

### 4.1 Limitations

This study has several limitations. The nonrandomized design limits causal inference, and the sample size reduced statistical power to detect modest between-group and regional trichoscopic differences. Follow-up was limited to 6 months, which may be insufficient to capture the full timeline of structural changes expected with systemic therapies for androgenetic alopecia. Loss to follow-up was observed, which is common in elective esthetic medicine studies, where participants may discontinue follow-up once subjective improvement is perceived or expectations are not met; however, attrition was balanced across treatment groups, reducing the likelihood of differential attrition bias. In addition, trichoscopic evaluations were restricted to predefined scalp regions and did not include global hair counts or whole-scalp imaging, potentially limiting sensitivity to early structural changes. A limitation of this study is that trichoscopic outcomes were derived from automated AI-based analysis, which may be less sensitive in high-density scalp regions due to hair overlap and clustering, and were not subjected to systematic manual correction. Finally, clinical severity was assessed using Hamilton–Norwood staging, which remains a subjective measure.

## 5 Conclusion

In this study, oral minoxidil at all three doses was associated with measurable short-term improvements in clinical severity in androgenetic alopecia over 6 months, while dutasteride did not demonstrate a statistically significant early change in clinical severity during the same period. Trichoscopic parameter responses were generally modest across all groups and did not demonstrate significant gains in total density, with structural changes observed to vary by region and dose. The divergence between clinical and microscopic findings suggests that, within short follow-up periods, potential treatment-related changes may be more readily reflected in clinical staging than in trichoscopic metrics. These findings highlight the potential value of integrating standardized clinical assessment with targeted, region-specific imaging to better characterize treatment response. Longer follow-up and larger, randomized studies will be important for advancing the evaluation of systemic therapies in androgenetic alopecia.

## Data Availability

The raw data supporting the conclusions of this article will be made available by the authors, without undue reservation.
