# Supplementary material for: Comparative efficacy of oral dutasteride and low-, medium-, and high-dose oral minoxidil: a six-month prospective trichoscopic and clinical study
Source: Front Med (Lausanne). 2026 Feb 16;13:1751116. doi: 10.3389/fmed.2026.1751116 (PMC12979936; doi:10.3389/fmed.2026.1751116)
Supplement: Supplementary file 1 [file Data_Sheet_1.PDF]

Appendix Figure 1. Participant Flow from Enrollment to Six-Month Outcomes across Treatment Groups

Appendix Figure 2. Six-Month Change (Δ) in Total Hair Density Across Regions and Treatment Groups

Appendix Table 1. Baseline demographic, clinical, and trichoscopic characteristics by treatment group

Variable	Dutasteride 0.5 mg	Minoxidil 5 mg	Minoxidil 2.5 mg	Minoxidil 1 mg
Sample size, n	14	17	19	17
Weight (kg)	84.8	87.8	80.4	78.1
Height (cm)	178.6	175.6	178.5	175.9
Body mass index (kg/m²)	26.3	28.4	25.2	25.2
Daily dose (mg)	0.5	5.0	2.5	1.0
Hamilton–Norwood stage (baseline)	2.4	3.4	3.5	3.3
**Fronto-parietal region**				
Total hair density (hairs/cm²)	173.5	160.1	171.9	189.8
Thick hair proportion (%)	58.6	60.2	60.6	53.0
Thin hair proportion (%)	18.6	17.1	17.3	21.0
Mean hair shaft caliber (µm)	56.6	56.9	55.2	53.8
Single follicular units (%)	46.9	50.7	46.5	46.4
Double follicular units (%)	33.3	34.5	34.6	33.5
Triple follicular units (%)	19.8	14.8	18.9	19.6
**Occipital region**				
Total hair density (hairs/cm²)	154.4	162.5	172.4	169.1
Thick hair proportion (%)	73.4	72.3	77.6	70.9
Thin hair proportion (%)	11.4	10.6	9.7	11.1
Mean hair shaft caliber (µm)	65.8	65.2	67.3	65.6
Single follicular units (%)	39.1	35.8	31.8	36.7
Double follicular units (%)	38.4	40.4	39.4	36.1
Triple follicular units (%)	22.4	23.8	28.8	25.5
**Temporal region**				
Total hair density (hairs/cm²)	121.4	122.5	125.8	134.6
Thick hair proportion (%)	74.1	74.8	77.2	74.0
Thin hair proportion (%)	13.1	11.5	10.1	13.3
Mean hair shaft caliber (µm)	64.6	66.0	65.7	65.5
Single follicular units (%)	49.1	41.5	38.8	41.6
Double follicular units (%)	35.6	41.3	42.6	39.7
Triple follicular units (%)	15.4	17.2	18.6	16.5

Appendix Table 2. Baseline demographic and trichoscopic characteristics by treatment group and completion status.

Treatment group	Dutasteride 0.5	Dutasteride 0.5	Minoxidil 5 mg	Minoxidil 5 mg	Minoxidil 2.5 mg	Minoxidil 2.5 mg	Minoxidil 1 mg	Minoxidil 1 mg
Completion status	**Completers**	**Non-completers**	**Completers**	**Non-completers**	**Completers**	**Non-completers**	**Completers**	**Non-completers**
Participants (n)	8	6	12	5	15	4	11	6
Weight (kg)	84.1 (26.6)	85.8 (14.2)	84.3 (14.8)	96.4 (15.6)	80.5 (10.9)	79.9 (9.1)	79.2 (14.2)	76.2 (19.6)
Height (cm)	178.4 (10.1)	179.0 (7.5)	176.3 (10.3)	174.2 (4.3)	179.1 (6.6)	176.0 (3.2)	176.1 (8.6)	175.5 (8.9)
BMI (kg/m²)	26.0 (5.8)	26.7 (3.0)	27.0 (3.3)	31.9 (6.0)	25.1 (3.1)	25.8 (2.9)	25.5 (3.8)	24.6 (5.2)
Hamilton–Norwood (baseline)	2.38 (0.74)	2.50 (0.55)	3.42 (0.90)	3.20 (1.64)	3.33 (0.82)	4.00 (2.00)	3.73 (1.35)	2.50 (1.52)
**Fronto-parietal region**								
Total density (hairs/cm²)	180.3 (38.0)	164.5 (25.7)	157.5 (40.3)	166.2 (36.8)	**182.3 (34.6)***	**132.8 (54.0)***	197.8 (39.7)	175.0 (28.2)
Thick hair (%)	58.5 (12.2)	58.8 (28.2)	55.7 (15.0)	71.0 (19.3)	61.8 (18.6)	56.3 (30.0)	**42.7 (20.2)***	**71.8 (14.7)***
Thin hair (%)	15.4 (9.0)	22.8 (22.6)	17.3 (10.1)	16.6 (21.1)	16.3 (14.9)	21.3 (18.2)	26.0 (18.4)	11.8 (7.6)
Mean caliber (µm)	55.3 (6.0)	58.3 (15.9)	54.8 (8.1)	62.0 (10.8)	55.3 (8.4)	54.5 (13.9)	**47.7 (10.0)***	**65.0 (10.8)***
Single FU (%)	47.1 (11.8)	46.7 (13.9)	50.5 (13.9)	51.2 (12.7)	44.1 (12.5)	55.5 (18.2)	44.6 (9.8)	49.7 (5.2)
Double FU (%)	32.1 (8.6)	34.8 (8.8)	34.8 (6.8)	33.8 (8.2)	34.7 (6.7)	34.0 (11.0)	33.9 (4.7)	32.8 (4.2)
Triple FU (%)	20.8 (8.2)	18.5 (8.1)	14.8 (8.8)	15.0 (7.5)	**21.1 (7.5)***	**10.5 (7.9)***	20.8 (8.6)	17.5 (5.2)
**Occipital region**								
Total density (hairs/cm²)	147.3 (27.3)	164.0 (35.9)	162.7 (24.2)	162.2 (25.9)	174.6 (31.0)	164.3 (43.8)	171.0 (31.7)	165.5 (31.8)
Thick hair (%)	70.8 (12.5)	76.8 (12.2)	72.3 (11.2)	72.4 (22.8)	78.9 (9.8)	72.5 (15.2)	67.5 (22.0)	77.0 (9.0)
Thin hair (%)	13.0 (5.0)	9.2 (5.9)	10.3 (6.2)	11.2 (9.0)	9.4 (6.5)	10.8 (7.9)	12.2 (7.0)	9.0 (4.9)
Mean caliber (µm)	64.3 (8.8)	67.8 (10.0)	64.5 (7.5)	67.0 (10.0)	67.2 (6.6)	67.8 (13.6)	64.5 (9.3)	67.8 (9.0)
Single FU (%)	40.9 (12.6)	36.8 (11.4)	32.8 (7.4)	43.0 (14.2)	32.1 (4.9)	31.0 (6.7)	35.0 (8.9)	39.8 (9.9)
Double FU (%)	38.3 (6.2)	38.7 (9.4)	42.4 (7.9)	35.6 (11.6)	38.1 (7.9)	44.0 (2.2)	37.4 (6.1)	33.8 (4.1)
Triple FU (%)	20.9 (9.9)	24.5 (12.5)	24.8 (7.1)	21.4 (7.9)	29.8 (6.9)	25.0 (7.9)	25.1 (9.7)	26.3 (10.1)
**Temporal region**								

Total density (hairs/cm²)	122.5 (24.6)	120.0 (23.7)	121.3 (20.1)	125.6 (44.5)	124.5 (29.6)	130.5 (27.6)	136.3 (20.7)	131.7 (32.7)
Thick hair (%)	73.5 (13.1)	75.0 (8.9)	76.2 (9.2)	71.6 (17.8)	78.1 (9.9)	73.8 (15.7)	71.6 (14.1)	78.3 (9.9)
Thin hair (%)	13.4 (5.6)	12.8 (6.4)	10.2 (5.8)	14.8 (12.3)	**9.6 (5.7)***	**12.0 (10.2)***	15.8 (9.2)	8.7 (7.2)
Mean caliber (µm)	65.0 (9.2)	64.2 (4.8)	65.8 (7.4)	66.4 (8.7)	65.3 (5.4)	67.0 (9.1)	63.1 (10.8)	69.8 (10.2)
Single FU (%)	49.1 (9.9)	49 (14.6)	40.3 (9)	44.4 (9)	36.6 (9.3)	47 (10.4)	40.7 (8.2)	43.3 (10.8)
Double FU (%)	34.7 (6.204837)	36.6 (11.360751)	42.5 (6.5)	38.2 (3.9)	43.4 (7.42)	39.5 (13.7)	39.9 (9.1)	39.3 (6.4)
Triple FU (%)	16.5 (6.9)	14.3 (6.5)	17.1 (5.8)	17.4 (9.7)	20 (9.7)	13.5 (4.9)	16 (7.7)	17.3 (7.6)

Baseline characteristics are presented as mean (standard deviation).

***p < 0.05**; exploratory non-parametric comparison using the Wilcoxon rank-sum test.
