# Supplementary figures and images for: Comparative efficacy of oral dutasteride and low-, medium-, and high-dose oral minoxidil: a six-month prospective trichoscopic and clinical study
Source: Front Med (Lausanne). 2026 Feb 16;13:1751116. doi: 10.3389/fmed.2026.1751116 (PMC12979936; doi:10.3389/fmed.2026.1751116)

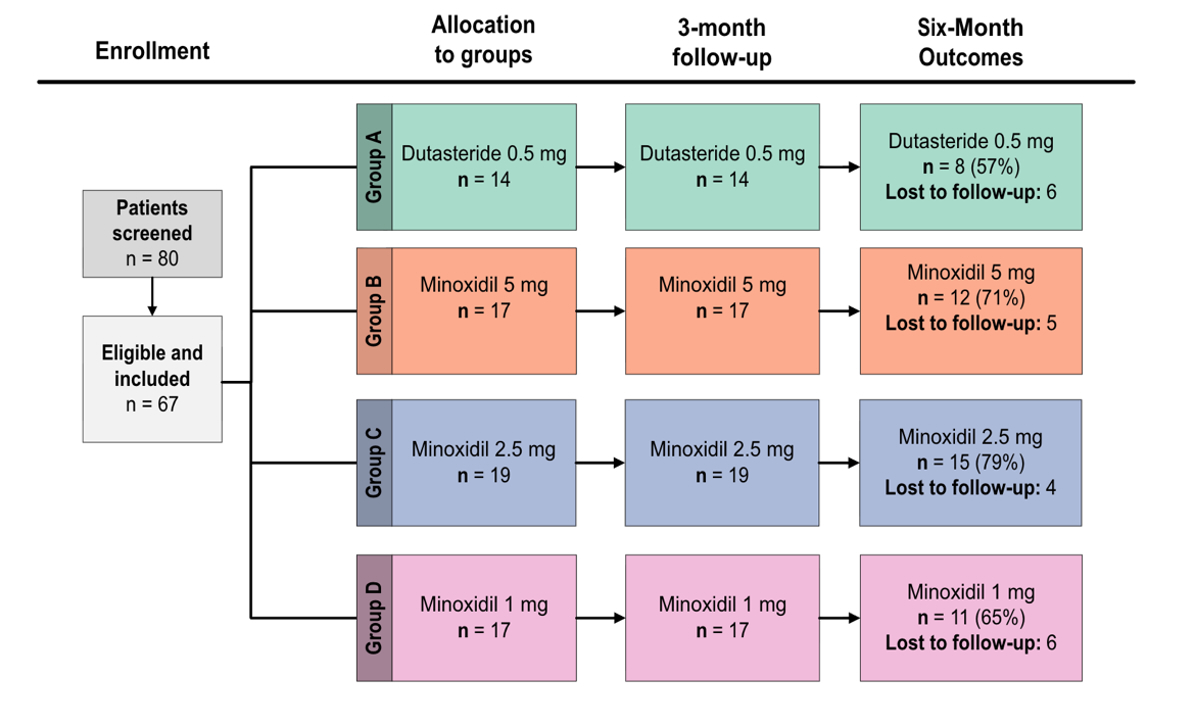

Supplement: Supplementary file 2 [file Image_1.JPEG]

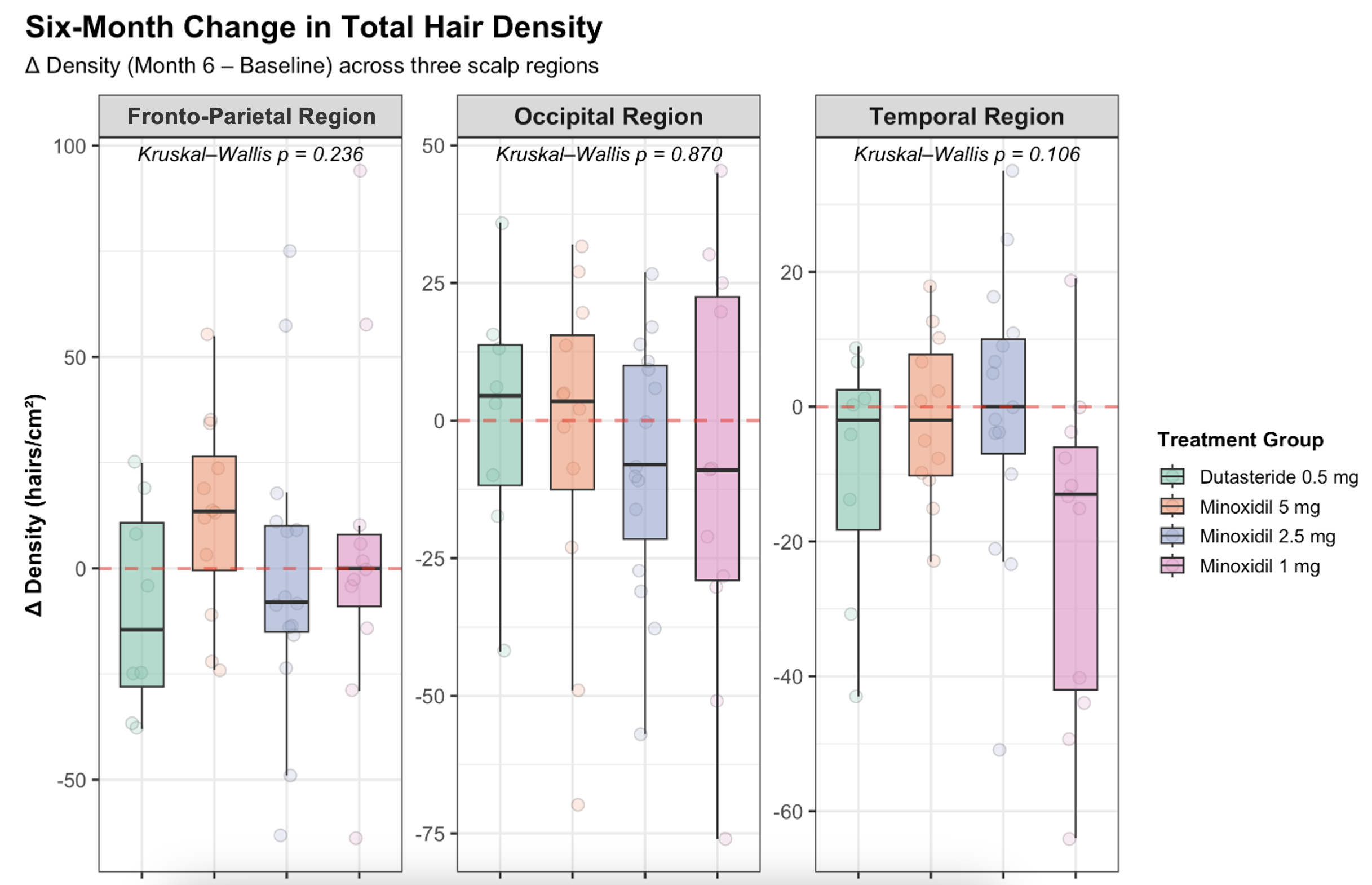

Supplement: Supplementary file 3 [file Image_2.JPEG]
